# Alterations in Localized Electrical Impedance Myography of Biceps Brachii Muscles Paralyzed by Spinal Cord Injury

**DOI:** 10.3389/fneur.2017.00253

**Published:** 2017-06-20

**Authors:** Le Li, Argyrios Stampas, Henry Shin, Xiaoyan Li, Ping Zhou

**Affiliations:** ^1^Department of Physical Medicine and Rehabilitation, University of Texas Health Science Center at Houston, Houston, TX, United States; ^2^TIRR Memorial Hermann Research Center, Houston, TX, United States; ^3^Department of Rehabilitation Medicine, The First Affiliated Hospital, Sun Yat-sen University, Guangzhou, China; ^4^Guangdong Work Injury Rehabilitation Center, Guangzhou, China

**Keywords:** electrical impedance myography, biceps brachii, muscle, multifrequency, spinal cord injury

## Abstract

This study assessed electrical impedance myography (EIM) changes after spinal cord injury (SCI) with a localized multifrequency technology. The EIM measurement was performed on the biceps brachii muscle at rest condition of 17 cervical SCI subjects, and 23 neurologically intact subjects as control group. The results showed that there was a significant decrease in muscle reactance (X) and phase angle (θ) at selected frequencies (i.e., 50 and 100 kHz) in SCI compared to control. There was no significant difference in muscle resistance (R) between the two groups. The anisotropy examination revealed that SCI group had a decreased anisotropy ratio in resistance. In addition, the multifrequency spectrum analysis showed a decreased slope of the log(freq)-resistance regression in SCI group when compared to healthy control. Findings of the EIM changes are related to inherit muscle changes after the injury. Since EIM requires no patient effort and is quick and convenient to conduct, it may provide a useful tool for examination of paralyzed muscle changes after SCI.

## Introduction

Individuals with cervical spinal cord injury (SCI) experience significant function impairment of their upper extremities, with subsequent challenges in activities of daily living (1). SCI predisposes survivors to muscle atrophy, contracture, and increased fat infiltration, which might be one of the causes of limited physical activity (2, 3). Muscle atrophy can impact the specific fiber type and is frequently accompanied by a slow muscle fiber to fast type fiber shift (4). The muscle changes may be also related to muscle disuse and higher levels of body fat leading to obesity and metabolic changes (5, 6). In addition, Biering-Sørensen and colleagues reviewed that there were muscle contractile changes besides the fiber-type transformation after SCI (7).

Various electrophysiological methods have been used to examine the paralyzed and spastic muscles after SCI. Abnormal electromyography (EMG) from paralyzed muscles of SCI subjects commonly include fibrillation and sharp waves (8, 9), spontaneous motor unit activity, motor unit loss, and enlarged motor unit action potentials (10–12), as a result of degeneration of spinal motoneurons and peripheral neuromuscular deterioration (13). These findings also provide evidence of alterations in muscle architecture and components after SCI. Bioelectrical impedance analysis (BIA) has been used to evaluate fat-free mass and total body water of SCI subjects (14). The whole body impedance results of BIA are limited in this application because selectivity is lost from individual muscles from paralyzed parts of body segments. In addition, one assumption of BIA method is that tissue-specific resistivity is constant for all body segments. However, tissue-specific resistivity has been shown to vary among body segments because of differences in tissue composition, hydration levels, and electrolyte concentrations (15). The results of whole body BIA could be impacted by the changes in the subject’s body gestures and joint angle position during the measurement, which further affects the repeatability and reliability of the results (16).

As a non-invasive bioimpedance-based technique, electrical impedance myography (EIM) is generally used to detect and quantify muscle health by sending high-frequency, low-intensity current into a discrete region of muscle tissue and measuring the consequent voltage (16). Three mainly used EIM parameters (17) include [1] resistance (R), representing the resistivity to current flow in the extracellular and intracellular fluids; [2] reactance (X), indicating how the current flow is affected by cell membranes and by the various fascia of the body; and [3] phase angle (θ), which is defined as θ = arctan (X/R). EIM has been used to reveal the changes in diseased muscles, such as in myopathies (18), radiculopathies (19), spinal muscular atrophy (SMA) (20, 21), Duchenne muscular dystrophy (DMD) (22), and amyotrophic lateral sclerosis (ALS) (23, 24). For example, the EIM findings from SMA demonstrated its usefulness of accurately categorizing patients between type 2 and type 3 (20). A longitudinal SMA study could also detect virtually static or no growth of active muscle maturation (21). In addition, EIM values were found to be correlated with standard ALS severity (25). Another important application of EIM is to investigate the anisotropy of muscle tissue which can be disturbed after pathological changes in neuromuscular diseases (26–28), since skeletal muscle is electrically anisotropic and current flow goes more easily in the longitudinal direction (parallel to the muscle fibers) than in the transverse direction. Neuromuscular diseases such as ALS might have impact on the anisotropy characteristics followed from muscle atrophy and fiber disorganization (29).

Electrical impedance myography has not yet been applied in assessing muscle health and anisotropy changes after SCI. The objectives of this study are to apply multifrequency local EIM technology to detect muscle impedance changes in individuals with SCI by comparing to age-matched healthy control subjects. To the best of our knowledge, this study represents the first effort of using EIM to examine SCI subjects, and the findings can provide useful information to understand architectural changes in muscles paralyzed by SCI.

## Materials and Methods

### Participants

A convenience sample of 17 chronic SCI subjects (4 females and 13 males, age 39.2 ± 13.0 years, duration of the injury 8.7 ± 7.3 years) with neurological injury levels from C2 to C6 and American Spinal Injury Association (ASIA) impairment levels of A–D, participated in this study. Twenty-three healthy subjects (11 females and 12 males, age 34.5 ± 7.3 years) were also recruited as control group. Clinical characteristics of the SCI subjects are summarized in Table 1. All the SCI subjects were recruited by using the outpatient clinic of TIRR Memorial Hermann Hospital (Houston, TX, USA). The neurologically intact subjects had no known history of any neurological disorder or neuromuscular disease and had normal strength and bulk of the biceps brachii muscle. All procedures of the study were performed in accordance with the Declaration of Helsinki, and this study was approved by the Committee for the Protection of Human Subjects of University of Texas Health Science Center at Houston and TIRR Memorial Hermann (Houston, TX, USA). All the subjects gave their written consent (or witnessed verbal consent if unable to write) before the experimental procedures.

**Table 1:** Clinical characteristics of the spinal cord injury subjects enrolled in this study.

Subject ID	Age	Gender	Years past injury	Neurological level	Motor level	ASIA impairment level
1	39	Male	3	C5	C5	D
2	49	Female	16	C3	C7	C
3	47	Male	10	C5	C5	C
4	50	Male	26	C6	C6	D
5	31	Female	1	C4	C7	C
6	38	Female	10	C6	C7	B
7	23	Male	9	C3	C3	A
8	51	Male	2	C6	C6	C
9	36	Male	16	C2	C2	A
10	29	Male	5	C3	C3	D
11	65	Male	2	C2	C2	C
12	60	Male	20	C5	C5	A
13	18	Male	4	C2	C2	D
14	36	Male	11	C5	C7	A
15	39	Female	10	C4	C7	B
16	31	Male	1	C4	C5	B
17	25	Male	2.5	C4	C7	D

Total *n* = 17	Average years = 39.2	13M and 4F	Average years = 8.7			

### Experiment

**Figure 1:**
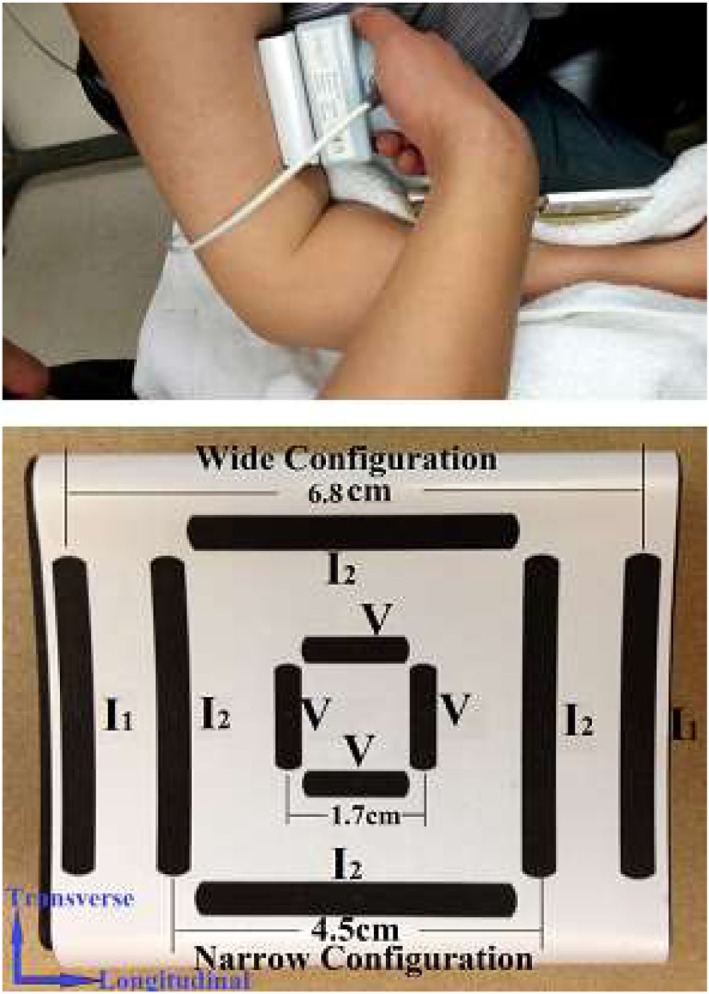
Experiment setup (top) and probe configuration (bottom). *I*: current injecting electrode; *V*: voltage measuring electrode.

The experiments were performed on the bilateral biceps brachii muscles of the SCI subjects and the dominant side of the healthy control subjects. The subjects were seated in a height-adjustable chair with the examined arm supported by the chair arm or by the examiner. The elbow joint was placed at 90° flexion and the shoulder at 45° abduction (Figure 1). The impedance measurements were performed using a handheld electrode array system (EIM1103, Skulpt Inc., MA, USA) that applies very low-intensity, high-frequency electrical currents at frequencies ranging from 1 kHz to 10 MHz, and then measures the consequent surface voltages. The sensor array was placed over the center of the muscle belly. The distance between the wider current electrodes [3.9 cm long, 0.4 cm width, indicated as *I*_(1)_] is 6.8 cm, the distance between the narrow current electrodes *I*_(2)_ is 4.5 cm, and the distance between the voltage bar electrodes (1.3 cm long, 0.4 cm width) is 1.7 cm (Figure 1). The EIM probe placement was longitudinal with the muscle fibers. There are three configurations of sending current and measuring voltage: configuration 1 is *I*_(1)_ wide pair sending current and *V* measuring at longitudinal direction, configuration 2 is *I*_(2)_ narrow pair sending current and *V* measuring at longitudinal direction, while configuration 3 is *I*_(2)_ narrow transverse pair sending current, and *V* measuring at according transverse direction. Sterile saline wipes (Hygea, PDI Inc., NY, USA) were applied to ensure the skin was sufficiently moist prior to performing impedance measurements.

### Clinical Evaluation

A physician who was blinded to the EIM results evaluated the SCI subjects with clinical scores such as ASIA impairment level, motor level, and the neurological injury level. The clinical characteristics are summarized in Table 1.

### Data Analysis and Statistics

The resistance (R), reactance (X), and phase angle (θ) (mean ± SD) were analyzed and reported. X, R, θ vs. applied frequency plots were also generated. R, X, and θ obtained at the frequency of 50, 100, and 200 kHz with wide longitudinal current electrodes configuration [*I*_(1)_, 6.8 cm distance] were used to examine the EIM changes in SCI compared to healthy control. Impedance data collected from narrow transverse and longitudinal current electrodes [two pairs of *I*_(2)_, 4.5 cm distance, Figure 1, bottom] were used for calculation of anisotropy ratio (AR) of each variable (X, R, and θ), which is defined as:

$$AR_{V}=\frac{V_{\text{Trans}}}{V_{\text{Long}}}$$

where *V*_Trans_ and *V*_Long_ represent impedance variables (X, R, or θ) collected in transverse and longitudinal directions, respectively (which means configuration 3/configuration 2, shown in Figure 1). AR impedance values were also compared across 50, 100, and 200 kHz frequencies. Another multifrequency parameter, the slope of the resistance-logarithm frequency [log(freq)-R slope], was calculated by performing a log transformation of applied current frequencies (selected from 8 to 10,000 kHz) and plotting the resistance points. The slope of a linear regression that fits those resistance points was taken as log(freq)-R slope. In order to minimize the impact by subcutaneous fat, a ratio of phase angle values (50/200 kHz) was calculated based on a previous DMD study (22). The ratio could better quantify the frequency characteristics of EIM with disease severity.

Electrical impedance myography from the SCI group’s left and right biceps showed variations and then were divided to stronger side and weaker side based on the physician’s motor exam and patient self-reports. Two ways analysis of variance (ANOVA) was used to compare each parameter of the three subject groups (i.e., healthy control group, stronger, and weaker side of SCI group) at different frequencies. The Bonferroni correction was used in pairwise comparison in the *post hoc* tests. Pearson correlation analysis was conducted between EIM parameters and clinical data of SCI. Statistics analysis was done using SPSS17 (IBM Inc., WA, USA). The significance level was determined as *p* < 0.05 for all statistical analyses.

## Results

### EIM Comparison

**Figure 2:**
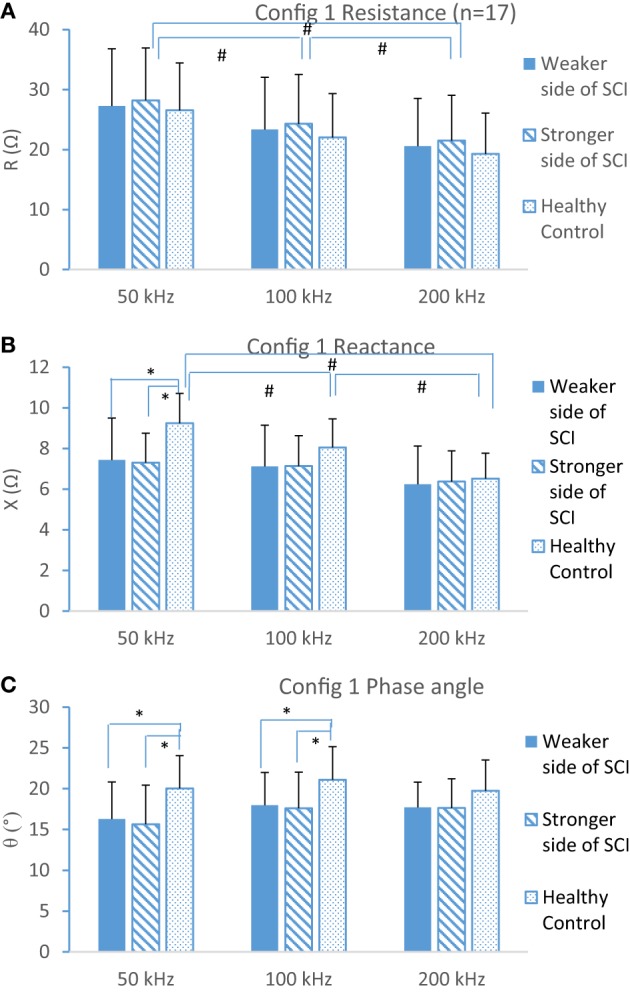
Resistance **(A)**, reactance **(B)**, and phase angle **(C)** at three different frequencies (50, 100, and 200 kHz) at weaker and stronger sides of spinal cord injury (SCI) and dominant side of healthy control (mean ± SD, * indicates *p* < 0.05 between groups, ^#^ indicates *p* < 0.05 from different frequencies).

Resistance (R), reactance (X), and phase angle (θ) from weaker and stronger sides of the SCI subjects and the control group at frequency of 50, 100, and 200 kHz were summarized in Figure 2. Two-way ANOVA results showed a significant interaction between frequency and subject group at X and θ, and *post hoc* test showed reactance at both the weaker side and stronger side was significantly lower (*p* < 0.001 and *p* < 0.002, respectively) than that from the control group at 50 kHz (Figure 2B). Similarly, phase angle at both the weaker side and stronger side was significantly smaller than healthy control (Figure 2C) at 50 and 100 kHz. However, there was no significant difference at resistance between groups (Figure 2A).

**Figure 3:**
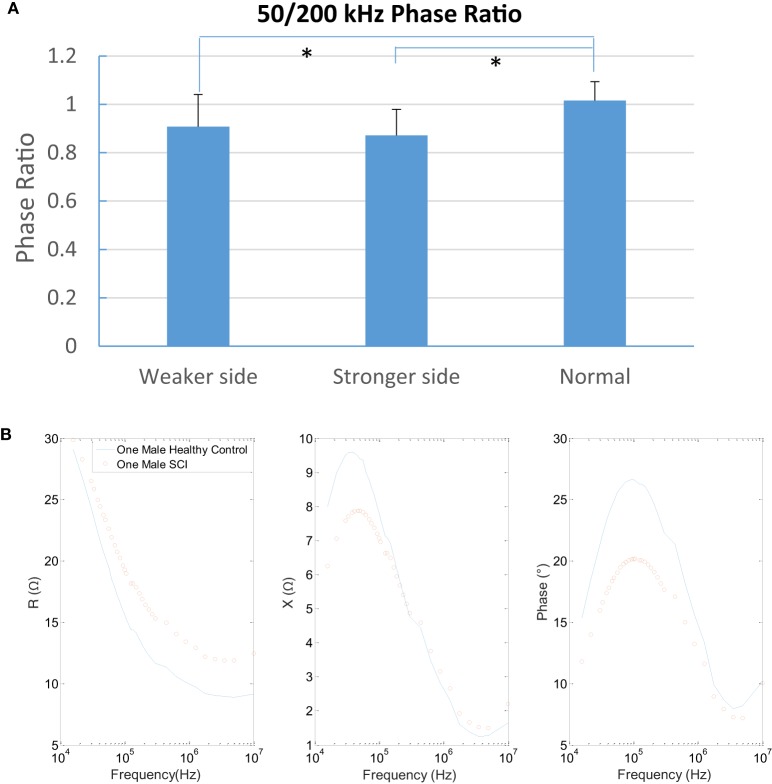
Comparison of 50/200 kHz phase ratio among three groups (mean ± SD, * indicates *p* < 0.05 between groups). A typical trial of spinal cord injury (SCI) and healthy control in multifrequency of three parameters.

Consider the frequency effects to each parameter, the resistance decreased with the frequency band at each subject group significantly (*p* < 0.05 at each group). However, the reactance from SCI subjects did not change with frequency (*p* > 0.05 at weaker and stronger sides). By contrast, in healthy control, group X significantly decreased with the frequency increasing (*p* < 0.05) (Figure 2B). The results showed that there was a significant difference between groups in phase angle ratio of 50/200 kHz (*p* < 0.0001) and *post hoc* test found the control group (ratio = 1.02) was larger than weaker side (ratio = 0.91, *p* < 0.001) and stronger side (ratio = 0.87, *p* < 0.001) (Figure 3). A typical relationship between the R, X, phase angle (θ), and the emitting frequency for a 31-year-old subject with SCI and a 28-year-old healthy male subject was also shown in Figure 3.

### AR Comparison

**Figure 4:**
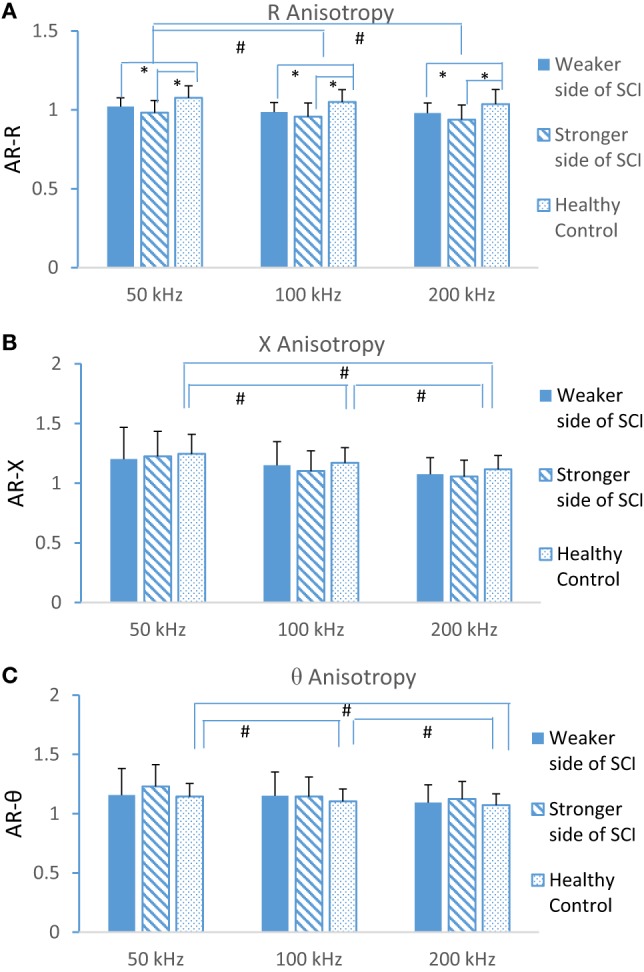
Anisotropy of resistance **(A)**, reactance **(B)**, and phase angle **(C)** at three different frequencies (50, 100, and 200 kHz) at weaker and stronger sides of spinal cord injury (SCI) and dominant side of healthy control (mean ± SD, * indicates *p* < 0.05 between groups, ^#^ indicates *p* < 0.05 from different frequencies).

Figure 4 shows the comparison of AR of R, X, and θ at three different frequencies. There was a significant difference of AR at resistance (Figure 4A) in subject groups, but not in reactance (Figure 4B) and phase angle (Figure 4C). The results revealed AR from the SCI group was significantly smaller compared to controls at R in selected frequencies. The frequency effect was significant for each of the AR parameters.

### Multifrequency Characteristics of EIM

**Figure 5:**
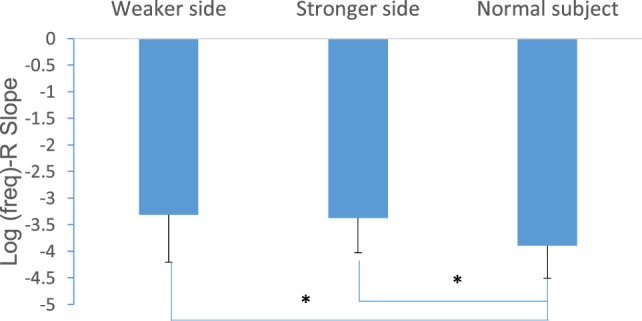
Comparison of the log(freq)-R slope between SCI and healthy control subjects.

Figure 5 shows a comparison of the slope of the linear regression describing the relation between R and log(freq) for the three groups. There was a significant difference in log(freq)-R slope between SCI (for both sides) and healthy control subjects (SCI weaker side slope = −3.31 ± 0.88, stronger side slope = −3.37 ± 0.65, the control slope = −3.89 ± 0.61, *p* < 0.05).

### Correlation between EIM and Clinical Parameters

Correlation analysis between EIM parameters such as X, phase angle, 50/200 kHz phase angle ratio, AR-resistance, and log(freq)-R slope to SCI clinical characteristics, such as time since injury and motor score was summarized in Table 2. The results showed the reactance (X) was significantly correlated with time post-injury (*p* = 0.035).

**Table 2:** Correlation analysis between clinical characteristics and electrical impedance myography (EIM) parameters.

EIM parameters										

Clinical data	X		Phase		Phase ratio		AR-R		Log(freq)-R slope	
	RHO	*p*	RHO	*p*	RHO	*p*	RHO	*p*	RHO	*p*
Time since injury	−0.513	0.035*	−0.08	0.756	0.149	0.569	0.197	0.448	0.455	0.067
Motor level	0.185	0.478	−0.08	0.747	−0.363	0.152	0.119	0.647	−0.23	0.374

*RHO, rank correlation coefficient*.

p: significant value with *<0.05.

## Discussion

We have demonstrated that, in SCI, multifrequency EIM patterns differ substantially from those in healthy control subjects at biceps brachii, especially the reactance and phase angle. The reactance of the SCI group was significantly lower than that from healthy controls at 50 kHz (Figure 2B). Reactance is related to the obstruction to current flow produced by the presence of capacitors (17). The smaller reactance in SCI is similar to previous EIM studies in aging, myopathy, and ALS (29–31). The decrease of reactance might be related to the disruption of cellular membrane integrity and injury (32). In addition, in animal study of ALS rat, the reduction of reactance was consistent with the ALS progression and also significantly correlated with the change in motor unit number estimation (MUNE) (33). Ahad and Rutkove also found the decreased reactance in animal study of sciatic injury (34). A tenable physiological interpretation of this condition is that muscle cell membranes have deteriorated severely in their ability to control the ionic flow (35) or the sarcolemma of individual myocytes are reduced in size and the membrane’s ability to momentarily store electrical charge is decreased (34). By using the sciatic crush rat model, Ahad and Rutkove correlated the EIM data and standard neurophysiologic parameters (such as nerve conduction, MUNE, and needle EMG) and found parallel changes in EIM to nerve motor conduction parameters (36). Therefore, EIM changes could be used to document the muscle cell membrane property changes after SCI as demonstrated in other populations. Rehabilitation interventions used to improve cellular membrane integrity or increase muscle myocyte size and number could be evaluated by EIM techniques that may help with the design of targeted therapies and/or outcome measures.

We found a significant smaller phase angle value in the SCI group at 50 and 100 kHz (Figure 2C). Phase angle represents the time shift of a sinusoid wave when passing through the muscle, which was previously used as a major outcome measure of EIM since it is less affected by the architecture and shape of the muscle compared with R or X (37). The smaller phase angle in the SCI group is in line with the data from a multicenter study of DMD children (38) and ALS subjects (23). Reductions in cell size and the presence of more connective tissue in the muscle also reduce the EIM phase angle (16). Another possible explanation might be that muscle disuse such as SCI causes type 1 fiber atrophy with a slow-to-fast fiber-type shift (2). Some muscles may undergo disuse myocyte atrophy and other muscles may be impacted by reinnervation (11). In addition, a smaller 50/200 kHz phase ratio was observed in this study (Figure 3), which may reflect the inherent property of muscle since electrical material properties of fat and muscle are distinct. Fat contributes similarly to the measured data at both 50 and 200 kHz, whereas muscle significantly contributing at 50 kHz and only minimally at 200 kHz. Thus by taking a ratio, it is possible to cancel out the effect of fat (22). Therefore, phase angle changes might be related to muscle cell atrophy and changes in fiber type in preserved paretic muscle of SCI survivors, which may reflect the subsequent effects of disuse, denervation, and/or reinnervation observed in electrophysiological studies (11, 39).

We did not reveal significant difference of resistance between the healthy control subjects and SCI subjects at selected frequencies, although the SCI group showed a larger trend of resistance. By using ultrasound, Gorgey and colleagues documented muscle atrophy of the wrist extensors that was 20–35% smaller in persons with tetraplegia compared with healthy controls (40). However, the impact of muscle size on impedance has remained a question and needs further discussion. For example, Rutkove and coworkers found the alterations in the EIM data in normal children were not simply an effect of increasing muscle mass and limb girth but rather a change in the structure and composition of the muscle itself (21). Another possible explanation for the resistance difference might be due to changes in subcutaneous fat thickness.

Significant decrease of anisotropy (ratio of transverse and longitudinal) of R in SCI at all three frequency values was found, when compared to those from healthy controls (Figure 4). This is similar to previous results from advanced ALS patients (28) and also similar to data from myopathy (29). Electrical anisotropy of skeletal muscle represents the inherit muscle fiber geometry within the muscle and the electrical current flowing more readily along muscle fibers than passing across them, since current perpendicular to fibers needs to go through more myocytes (26). After SCI, the survivors may suffer the loss of muscle fibers and associated fatty infiltration of tissue, leading to a general reduction in anisotropy since the muscle fibers are replaced by isotropic tissues such as inflammatory cells, connective tissues, and fat (29). Reduction of anisotropy was also observed in bovine muscle when it undergoes tissue disruption (27). However, neurological diseases may not always lead to decreases in anisotropy. For example, ALS patients in the early stage demonstrated an increased reactance anisotropy that may be due to reinnervated muscle fibers (28). Some metabolic myopathies might only cause subtle changes in muscle fiber size and structure and are associated with minimal deposition of endomysial connective tissue or fat, which would limit the ability of EIM to detect abnormalities. In addition, it is possible that unusual anisotropy results are simply due to the variation of direction of fiber arrangements within the muscle, while the fiber properties themselves are not changed (17). With further advances in measurement techniques, e.g., muscular ultrasound imaging, it may be possible to help understand the mechanism of alterations of electrical anisotropy and put this distinctive property of muscle to effective practical use and to differentiate the muscle changes from neurogenic and myopathic diseases.

In this study, both the EIM and electrical anisotropy showed a frequency dependency. Studies have shown that utilizing multifrequency measures may be more sensitive to disease status and progression over time (21, 22). With the frequency changes in the injecting current, the relative weights of resistive (fluid) and reactive (membranes) contributions to the total impedance are shifted, i.e., the cell membranes are modeled like the capacitor in an electrical circuit that very high frequencies make nearly no contribution of reactance to impedance (37). The changes in the frequency spectrum of the EIM may be due, in part, to reductions in myocyte size and number. This will lead to a decrease in the reactance, because in advanced chronic disease, myocytes are replaced with connective tissue and fat, and these changes in muscle composition will distort the normal spectra. Our results showed that the healthy control subjects had a larger log(freq)-R slope than the SCI subjects (Figure 5), which indicates that with increased frequencies the resistance of healthy control muscles decreased precipitously compared to paralyzed muscles. This finding is similar to a previous EIM clinical study on SMA children (20) in which they also observed a relatively small slope in the SMA group compared to the age-matched control group which may be caused by muscle fibrosis and fatty infiltration. The findings of this study support that using multifrequency EIM may potentially be clinically useful for extracting muscle-specific parameters for SCI survivors (16).

There are some cautions to be taken when interpreting the results of this study. We used the localized EIM technique that has been validated with high reproducibility (41). The probe size fits the biceps brachii muscle and the distance between current and voltage electrodes when calculating anisotropy is 1.4 cm. The optimal electrode distance between injecting current and measuring voltage to use is likely dependent on details of the skin–subcutaneous fat layer thickness of measured muscle (28, 42). With increasing fat and adipose tissue, a greater inter-electrode distance may be required to allow the current to go through the subcutaneous fat layer and then to effectively measure the anisotropy. This may be a plausible explanation why the anisotropy in this study is much lower than that measured on bare bovine muscles (27). A future dedicated study assessing the relationship between inter-electrode distance, subcutaneous fat layer thickness (could be measured from ultrasound), and the measured EIM changes in SCI will be useful. In addition, we referred to previous published studies that demonstrated the localized EIM’s good reliability and repeatability on human subject (35, 41). It may be better to have body mass index of SCI subjects in the future to correlate with EIM that can further verify the body size or limb girth effects. Second, this EIM comparison of patients with neurologically intact control subjects is considered as only the first step to demonstrate the feasibility of applying EIM in muscle health evaluation and to identify parameters that can represent clinically evident changes in muscle tissue of SCI survivors. An attempt was made to correlate the X, phase angle, 50/200 kHz phase ratio, AR-resistance, and log(freq)-R slope to SCI clinical characteristics, such as time since injury and motor score. We only find statistically significant relationship between X and time since injury (*p* = 0.035, Table 2). Therefore, the changes in EIM alone do not fully explain the mechanism of complex neuromuscular changes after SCI. The major finding of this EIM study is the difference between paralyzed and matched muscles, and EIM could provide a convenient approach to quantifying paralyzed muscle changes. The combination of other imaging techniques (i.e., musculoskeletal ultrasound), electrophysiological techniques such as MUNE (25) and quantitative motor unit action potential analysis is warranted to provide a comprehensive picture to aid in the understanding of denervation, reinnervation, and/or other neuromuscular alterations due to SCI.

In summary, we demonstrated that it is feasible to apply EIM in muscle health evaluation in persons with cervical SCI. Our results revealed reductions in reactance and phase angle in SCI group compared to healthy control which might be related to the changes in muscle inherent and structural property alterations after injury. In addition, we also observed that the AR of resistance was smaller which might be related to loss of muscle fibers and fat infiltration. These findings might provide novel information for understanding the muscle changes after SCI. The multifrequency EIM technique can further strengthen EIM as a potential tool for the non-invasive assessment of paralyzed muscles.

## Ethics Statement

All procedures of the study were performed in accordance with the Declaration of Helsinki, and this study was approved by the Committee for the Protection of Human Subjects (CPHS) of University of Texas Health Science Center at Houston and TIRR Memorial Hermann (Houston, TX, USA). All the subjects gave their written consent (or witnessed verbal consent if unable to write) before the experimental procedures.

## Author Contributions

LL performed experiment, data analysis and interpretation, and wrote the first draft of the manuscript. AS provided clinical support for the study including SCI subject recruitment and evaluation, helped data analysis and interpretation, and revised manuscript. HS and XL provided technical support for the study, helped with experiment and data analysis, and revised manuscript. PZ oversaw different aspects of the study.

## Conflict of Interest Statement

The authors declare that the research was conducted in the absence of any commercial or financial relationships that could be construed as a potential conflict of interest.

## Abbreviations

ASIA, American Spinal Injury Association; EIM, electrical impedance myography; SCI, spinal cord injury.
